# The Development of VegEze: Smartphone App to Increase Vegetable Consumption in Australian Adults

**DOI:** 10.2196/10731

**Published:** 2019-03-27

**Authors:** Gilly A Hendrie, Genevieve James-Martin, Gemma Williams, Emily Brindal, Ben Whyte, Anna Crook

**Affiliations:** 1 Health & Biosecurity Commonwealth Scientific and Industrial Research Organisation Adelaide Australia; 2 SP Health Co Pty Ltd Sydney Australia

**Keywords:** mHealth, mobile applications, vegetables, adult

## Abstract

**Background:**

Poor-quality dietary patterns are often characterized by inadequate consumption of fruits and vegetables. Changing dietary behavior is difficult, and although it is often clear what needs to change, how to enact change is more difficult. Smartphones have characteristics that may support the complexity of changing dietary behavior.

**Objective:**

This paper describes the iterative process of developing a theory-based smartphone app called VegEze that aimed to increase vegetable consumption.

**Methods:**

To upscale, reach target users, and create a user-friendly end product, a collaborative research-industry partnership was formed to build the app over a 20-week period. The Integrate, Design, Assess, and Share framework was used as a scientific basis to guide the development. The behavior change wheel was also used as a theoretical grounding in combination with other theory-based strategies, such as self-monitoring, social comparison, and gamification—which have all been shown to be successful in dietary change or digital health interventions. We conducted 1 consumer survey (N=1068), 1 usability testing session (N=11), and a pilot effectiveness and usability trial (N=283) to inform the design of the app.

**Results:**

The target behavior for the app was defined as *having 3 different types of vegetables at dinner.* The perceived achievability of this target behavior was high; 93% of respondents (993/1068 users) felt they were *likely* or *very likely* to be able to regularly achieve the behavior. App features that users wanted included the following: recipes and meal ideas (876/1068, 82% of users), functionality to track their intake (662/1068, 62%), and information on how to prepare vegetables (545/1068, 51%). On the basis of importance of self-monitoring as a behavior change technique (BCT) and its rating by users, the vegetable tracker was a core feature of the app and was designed to be quick and simple to use. Daily feedback messages for logging intake and communicating progress were designed to be engaging and fun, using friendly, positive language and emoji icons. Daily and weekly feedback on vegetable consumption was designed to be simple, informative, and reinforce monitoring. A creative team was engaged to assist in the branding of the app to ensure it had an identity that reflected the fun and simple nature of the underlying behavior. The app included 16 BCTs, most of which were from the goals and planning subsection of the BCT taxonomy.

**Conclusions:**

Combining a theoretical framework with an industry perspective and input resulted in an app that was developed in a timely manner while retaining its evidence-base. VegEze is an iOS app currently available in the App Store, and the overall impact of the VegEze app will be evaluated in an uncontrolled, quantitative study.

**Trial Registration:**

Australian New Zealand Clinical Trials Registry ACTRN12618000481279; http://www.anzctr.org.au/TrialSearch.aspx?searchTxt=ACTRN12618000481279 (Archived by WebCite: at http://www.webcitation.org/769oG9EaA)

## Introduction

### Background

Poor diet quality is one of the most important modifiable risk factors for chronic disease [1]. Poor-quality diets are generally characterized by inadequate consumption of fruits and vegetables, and overconsumption of unhealthy, energy-dense, discretionary foods [2]. A diet that contains plenty of vegetables, including a variety of types and colors, can provide a range of beneficial nutrients that may help to reduce the risk of obesity and some chronic diseases [3]. Despite the known benefits of increasing vegetable consumption, intake remains low [4,5]. For example, in Australia, about 95% of adults do not meet the recommended intake of vegetables [5], which means that they are missing out on essential vitamins, minerals, and dietary fiber that vegetables can provide.

Population-level nutrition and obesity prevention interventions have had small to modest success in improving diet quality [6-8]—most likely because dietary behavior change is difficult and multifaceted. Although, it is often clear what needs to change, for example eat more vegetables, how to action changes is more difficult [9]. The interplay between individual-level (eg, willpower and motivation), household-level (eg, availability and finances), and community-level factors (eg, accessibility to fresh food and social norms) are all likely to affect dietary change [10].

Smartphones have characteristics which may support the complexity of changing dietary behavior. For example, smartphones are increasingly ubiquitous, have the ability to reach individuals at nearly any time or place, can be highly interactive, can deliver information in a way that is engaging and rewarding, and provide timely feedback [11]. Tailored feedback can also grow with user inputs [12], creating a personalized experience, which may encourage extended engagement and success with an intervention [13]. Therefore, smartphone-based behavior change interventions have the potential to be effective and also accepted by individuals. They may also serve as a cost-effective and scalable way to deliver behavioral nutrition interventions to a large audience. However, to have the greatest likelihood of success, these interventions also need to utilize existing scientific knowledge and theory [12,14].

An explosion of healthy eating smartphone apps has occurred in recent years (eg, MyFitnessPal, Lifesum, Lose It!, and Easy Diet Diary). However, many commercial apps are not scientifically developed, based on behavioral theory or evidence, and have yet to undergo rigorous evaluation [11,15]. Regardless of this, high and consistent downloads suggest they have significant reach and appeal, at least in the short term [16]. Apps are also being developed and used by the research community, but unfortunately the pace of development and evaluation in research means that the temporal lag limits their potential impact [17]. Despite contrasting times from conceptualization to market, both scientific and commercial entities want to develop engaging and effective apps and working together could result in better end products.

### Objectives

This paper describes the iterative development process to build a mobile app that considers both the scientific and commercial validity and that targets a complex and critically important dietary behavior—increasing vegetable consumption. The Integrate, Design, Assess, and Share (IDEAS) framework was used as a scientific basis to guide the development of the app [18]. The IDEAS framework is a flexible approach for developing digital interventions and is used here to provide a detailed description of the journey of building VegEze—an app to increase vegetable variety and consumption.

## Methods

### Ethics Approval

The development and evaluation of the app was approved by the Commonwealth Scientific and Industrial Research Organisation (CSIRO) Health and Medical Human Research Ethics Committee Low Risk Review Panel (LR13/2017) and registered with the Australian New Zealand Clinical Trials Registry (ACTRN12618000481279).

### Overview

**Figure 1:**
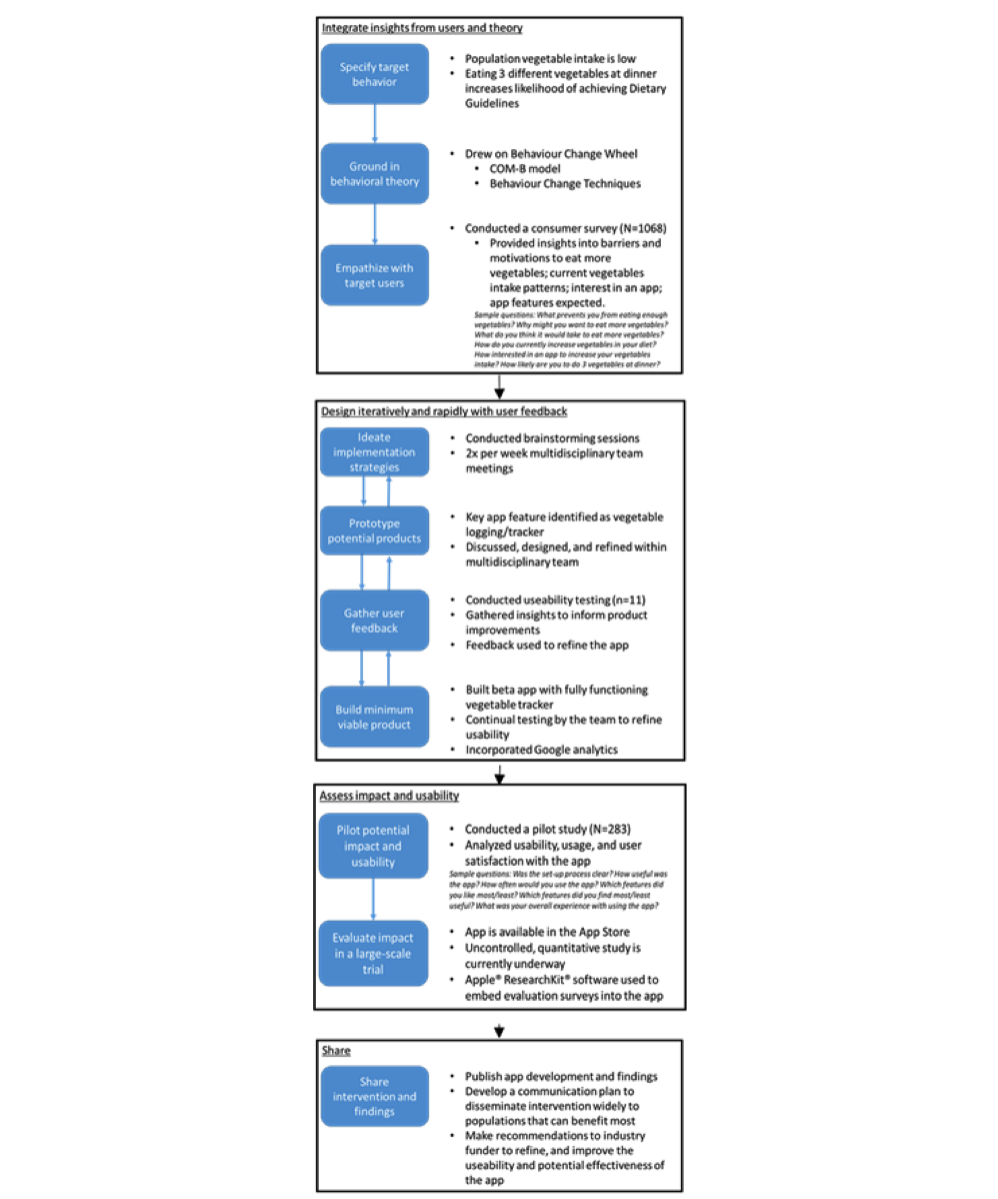
The application of the IDEAS (Integrate, Design, Assess, and Share) framework to the development of the VegEze smartphone app. Due to project requirements, the first 3 phases of part 1 of this study took place in a different order to original IDEAS framework. COM-B: capability, opportunity, and motivation to engage in the behavior.

The development of the VegEze app was conducted using the IDEAS framework. The IDEAS framework brings together a combination of approaches necessary for the development of effective technologies. The framework draws on behavioral theory, design thinking, user-centered design, rigorous evaluation, and dissemination—which have all been shown to be important for digital health interventions [18]. The framework outlines a step-by-step process for integrating these approaches, and although depicted as a linear process, it is intended to be iterative, and stages should be revisited as required during the development process [18]. The framework also provides theory-based behavioral science strategies to support the intervention design. Figure 1 is an adaptation of the IDEAS framework flowchart [18], providing an overview of the processes that were used in the development of the VegEze app.

### Part 1: Integrating Insights From Users and Theory

#### Procedure

The first 3 phases of the IDEAS framework were designed to gather insights from users and behavioral theory to focus the intervention process around a specific and measurable target behavior.

##### Phase 1: Specify Target Behavior

Specifying the target behavior was, in the first instance, an evidence-based decision drawing on existing data and literature, which was later tested with potential users before proceeding with the app development. To determine the degree of the problem, which is inadequate consumption of vegetables in the Australian population, we examined data from the Australian National Nutrition and Physical Activity survey. This is the latest nationally representative survey of dietary intake available in Australia [5]. The complex sampling method and design of this survey means we are able to make estimates of intake for the Australian population based on the data collected from a sample of 9341 adults [19]. These data provided estimates of the percentage of the population meeting the recommended vegetable intake targets as prescribed by the Australian Dietary Guidelines [3].

To more precisely define a specific target behavior within the context of increasing vegetable consumption, secondary data analysis of the CSIRO Healthy Diet Score survey was performed. The CSIRO Healthy Diet Score is an online short food survey that has collected data from over 198,000 Australians [2]. This survey asks about food group intake such as fruits and vegetables, as well as a range of food habits including *how often does your evening meal contain 3 or more different vegetables?* Secondary analysis explored the association between this specific behavior and individuals’ total vegetable consumption and likelihood of meeting the Australian Dietary Guideline targets.

##### Phase 2: Ground in Behavioral Theory

The development of the app also drew on the behavior change wheel (BCW) as a theoretical grounding [20,21] in combination with other theory-based strategies, such as self-monitoring, social comparison [22-24], and gamification [25-27]—which have all shown to be successful in either dietary change or digital health interventions. The BCW is an integrative guide for understanding behavior change and is a synthesis of many previously published theoretical frameworks from this field. Central to the BCW is the capability, opportunity, motivation, and behavior (COM-B model) that recognizes that for a behavior to occur, an individual must have the capability, opportunity, and motivation to engage in the behavior (COM-B). The first step of the BCW process is to specify the target behavior (similar to phase 1 described here). Then, subsequent steps identify what needs to change to perform this target behavior. To help quantify this and prioritize features of the app, we administered the COM-B self-evaluation questionnaire in a sample of potential users [20].

The app also drew on theory-based behavior change techniques (BCTs). The final version of the app was independently coded by 2 trained research assistants for inclusion of techniques from the 93 BCTs taxonomy [23]. This coding was done at the end of the development process to reflect the techniques employed in the final version of the app.

##### Phase 3: Empathize With Target Users

We conducted an online consumer survey with potential users to understand how the identified target behavior (eat 3 different vegetables at dinner) was received by potential users, and to elicit perceptions about performing this behavior.

###### Participants

Potential users were recruited via email from a database of individuals who had previously participated or expressed an interest in participating in online nutrition and health-related surveys or programs. An email was sent to 9900 individuals on the database and 1068 individuals completed the survey within 24 hours. These participants were majoritively female (84% female) with an average age of 56 years (range 20-90 years).

### Part 2: Design Iteratively and Rapidly With User Feedback

#### Procedure

##### Phase 4: Ideate Creative Implementation Strategies

To upscale, reach target users, and create a user-friendly end product, a collaborative research-industry partnership was formed which brought a multidisciplinary team together to build the app over a 20-week build phase. The team was led by a product development manager (from industry) and nutrition scientist (researcher), and included research dietitians, behavioral scientists, product developers, and software engineers. The multidisciplinary project team met regularly throughout the development process to ensure a rapid build phase. The ideation phase also focused on translating insights from users and theory into features of the app. The process of brainstorming ideas for the app was highly iterative and incremental.

The app was built using the principles of agile software development which supports a *minimum viable product* approach. The development team had 7 2-week sprints (14 weeks total development) and aimed to release usable features at the end of each sprint for the broader project team to review and then test with users.

##### Phase 5: Prototype a Potential Product

The build started by prototyping different ways to track vegetable intake. On the basis of the importance of self-monitoring as a BCT and its rating by potential users in the consumer survey (a tracker for vegetable intake was the second highest rated feature that users expected and highest in terms of a functionality feature), this feature was going to be a core component of the app. In addition to communicating progress, a tracker can be used to communicate and reinforce the specific target behavior.

##### Phase 6: Gather User Feedback on the Prototype

Initial user testing was conducted in a small sample of people, who were not known to the project team. Members of the development team approached individuals and asked them to complete 4 tasks on the prototype provided. The individuals were observed using the prototype app as they were asked to: add cauliflower, sweet potato, and corn to dinner; add capsicum to breakfast; add salad for lunch; and add carrots for a snack.

###### Participants

A total of 11 people were approached within a local cafe and asked to participate in the initial user testing session. They were asked to use to the app to do the 4 tasks and provide feedback on their experience. The perceived age of the participants was 25 to 45 years (demographic information was not asked to maintain anonymity).

##### Phase 7: Build Minimum Viable Product

The beta version of the app was developed with fully functional versions released and tested iteratively by the project team, with feedback provided to the project lead of the development team at regular intervals. To ensure the effectiveness and usability of the app could be assessed, evaluation surveys were designed and embedded into the app using Apple ResearchKit software. The length and usability of these surveys were tested as part of this phase as well. The beta version of the app focused on the onboarding process, refining the vegetable tracker and user experience, developing a framework for providing feedback, and the visual branding. In addition, a creative team was engaged to assist in the branding of the app to ensure it had an identity that reflected the fun and simple nature of the underlying behavior.

### Part 3: Assess Effectiveness and Usability

#### Procedure

##### Phase 8: Pilot Potential Effectiveness and Usability

The aim of this phase was to conduct a small-scale evaluation to test the potential effectiveness of the app and to inform final refinements as required. In this phase, it was also important to understand potential of usage of the app in terms of frequency and duration of use, as well as user satisfaction with the app. Potential users were invited to use the app for 3 days, providing feedback through 3 evaluation surveys that included 1 immediately post download, 1 after 24 hours of use, and 1 after 3 days of use. On the basis of the feedback and experience of the participants, a list of suggestions for improvements was compiled. The list of suggestions was discussed among the project team and prioritized before it was implemented into the final version of the app.

###### Participants

A new sample of potential target users, recruited through the same database described above, was invited to download the beta version of the app. A total of 553 participants registered their interest in the pilot study. Of these, 311 downloaded the app (311/553, 56% of those interested) and 283 completed the baseline survey (283/311, 91% of those who downloaded the app). Participants used the app for about 24 hours and were asked to complete a second survey (post download survey, n=146), followed by a final survey about 3 days after download (post pilot survey, n=103). The sample that completed the baseline survey was largely female (84%), with mean age 48 years, and 46% were overweight or obese.

## Results

### Part 1: Integrating Insights From Users and Theory

#### Phase 1: Specify Target Behavior

Data from the latest Australian National Nutrition survey suggest that less than 4% of adults consume enough vegetables to meet the Australian Dietary Guidelines [5]. The Australian Dietary Guidelines and Go for 2 & 5 campaigns encouraged the population to “enjoy plenty of vegetables”, “increase consumption”, and include “different types and colours” with a prescription for total daily recommended servings [7]. However, in the context of behavior change, the advice within this literature broadly addresses the problem of inadequate vegetable consumption, without identifying a highly specific target behavior.

Data from the CSIRO Healthy Diet Score survey showed that respondents reporting “always” having 3 different types of vegetables at their evening meal had higher overall vegetable consumption relative to other frequencies. These people were also more likely to meet the recommended daily intakes [28]. Review of other literature also suggests that serving a variety of vegetables can help in selecting a healthier meal [29] and is an effective strategy to increase vegetable consumption in a single meal [30]. On the basis of the CSIRO Healthy Diet Score data and other acute studies, the target behavior for the app was defined as *having 3 different types of vegetables at dinner*. This is a novel, specific, and an actionable behavior that is associated with an increased likelihood of the desired outcome—that is increased vegetable consumption. In addition, it is measurable, easy to self-monitor, and has the potential to produce a cascade benefit, that is improving vegetable consumption at other meal times. Therefore, the initial target behavior was to *eat 3 different vegetables at dinner*.

#### Phase 2: Ground in Behavioral Theory

The app draws on the BCW framework, and the COM-B self-evaluation questionnaire was administered as part of the online consumer survey to elicit reasons associated with individuals’ COM-B. When asked what they thought it would take to increase vegetable intake, 37% of responses from survey participants were reasons associated with their capability (eg, have better planning skills, cooking skills, and knowing how to eat more vegetables), 33% were for reasons associated with motivation (eg, developing better plans and a habit of eating more vegetables, feeling like I want to eat more vegetables), and 30% were reasons associated with opportunity (eg, having more time and triggers to prompt me to eat more vegetables). Therefore, respondents reported needing help in all areas of behavior change according to the COM-B theory (Table 1).

**Table 1:** Most common reasons reported for wanting to eat more vegetables from the consumer survey (N=1068). Survey question: “Why might you want to eat more vegetables?”

Response to survey question	Users, n (%)
To be healthier	822 (77)
Increase nutrient intake	726 (68)
Increase fiber intake	662 (62)
Want to lose weight	555 (52)
Feel better	491 (46)
Meet recommendations	267 (25)
Feel like I should	160 (15)
Doctor or health professional told me to	32 (3)
Friends/family eat more than I do	11 (1)

#### Phase 3: Empathize With Target Users

The online consumer survey aimed to understand how the target behavior (eat 3 different vegetables at dinner) was received by potential users, and to elicit perceptions about performing this behavior. Respondents were generally health conscious and health literate, with 94%-99% reporting that they felt it was “important” or “very important” to eat enough and a wide variety of vegetables each day, and 71% correctly identifying the daily recommended number of serves of vegetables. However, 66% of this sample still believed they would like to eat more vegetables, and this was largely for health-related reasons (Table 2).

In addition, 56% of respondents were “interested” or “very interested” in an app to specifically help with achieving the target behavior of eating 3 types of vegetables at dinner (Table 3). Interestingly, this was similar to those interested in the more traditional intervention target of “increasing vegetable intake” (55%). In the context of the app and perceived achievability of this target behavior, 93% of respondents felt they were “likely” or “very likely” to be able to regularly achieve the behavior, and most (68%) thought they would be able to do it for 30 days or more (Table 3).

In addition to administering the COM-B self-evaluation questionnaire, the survey respondents indicated their preferences for features in an app and their current vegetable eating behaviors. App features that users reported to want included recipes and meal ideas (82% of users), functionality to track their intake (62%), and information on how to prepare vegetables (51%, Table 3).

Most people reported to consume vegetables at their dinner meal (98% of respondents), but many also included vegetables with lunch (85%). Consuming vegetables at breakfast (11%) or as a snack (19%) was less common. The most common ways participants indicated eating vegetables in their meals were by including vegetables in dishes such as stir fries (48%), salads (39% as a side salad and 38% as a main), and 32% reported *hidden* within a dish such as spaghetti bolognaise. In addition, 66% of respondents reported that they *mix it up* and consume vegetables in a variety of ways. This information was used to guide the development of recipes and meal ideas.

**Table 2:** Most common reasons from the Capability, Opportunity, and Motivation to Engage in the Behavior Self-Evaluation Questionnaire about what it would take to increase vegetable intake from the consumer survey (N=1068). Survey question: “What do you think it would take to increase your vegetable intake?”

Response to survey question	Users, n (%)
Having better planning skills	150 (14)
Developing better plans for eating more vegetables	128 (12)
Developing a habit for eating more vegetables	107 (10)
Having more time	96 (9)
Having better cooking skills	85 (8)
Knowing how to eat more vegetables	75 (7)
Having more triggers to prompt me to eat vegetables	64 (6)
Developing greater will power	53 (5)
Feeling that I want to eat more vegetables	53 (5)

**Table 3:** Evaluation questions about the proposed app from the consumer survey (N=1068).

Survey question		Users, n (%)
**How interested are you in an app to help you to eat 3 types of vegetables at dinner?**		
	Very interested	299 (28)
	Interested	299 (28)
	Neutral	224 (21)
	Not interested	96 (9)
	Not at all interested	117 (11)
**How long do you think you could do this for?**		
	Less than 7 days	53 (5)
	7 days	96 (9)
	14 days	107 (10)
	21 days	85 (8)
	30 days or more	726 (68)
**What features would you like to see in an app to increase vegetable intake?**		
	Recipes	726 (82)
	Tracker	662 (62)
	How to info	545 (51)
	Weekly reports	459 (43)
	Info on guidelines	417 (39)
	Health info	342 (32)
	Challenges	310 (29)
	Rewards	235 (22)
	Engage family	150 (14)
	Photo gallery	96 (9)
	Social sharing	64 (6)
	Leaderboard	43 (4)

### Part 2: Design Iteratively and Rapidly With User Feedback

#### Phase 4: Ideate Creative Implementation Strategies

The multidisciplinary project team met twice per week throughout the 20-week development process that allowed us to generate and refine ideas and implement behavior change strategies within features that target users had reported to expect from the app. This was done collaboratively to retain the scientific evidence base while also maximizing the user experience.

At the end of each of the 7 development sprints, usable features were released, reviewed, and tested by the project team. From the fourth sprint, we started to release features to test with users. By using this approach, we were continually able to provide feedback to the development team and facilitate a rapid assembly of the app.

#### Phase 5: Prototype a Potential Product

Consumer feedback indicated that a large proportion of users consumed vegetables at lunch as well as dinner, so the project team decided that the tracker would allow vegetables to be tracked at all meals across the day.

**Figure 2:**
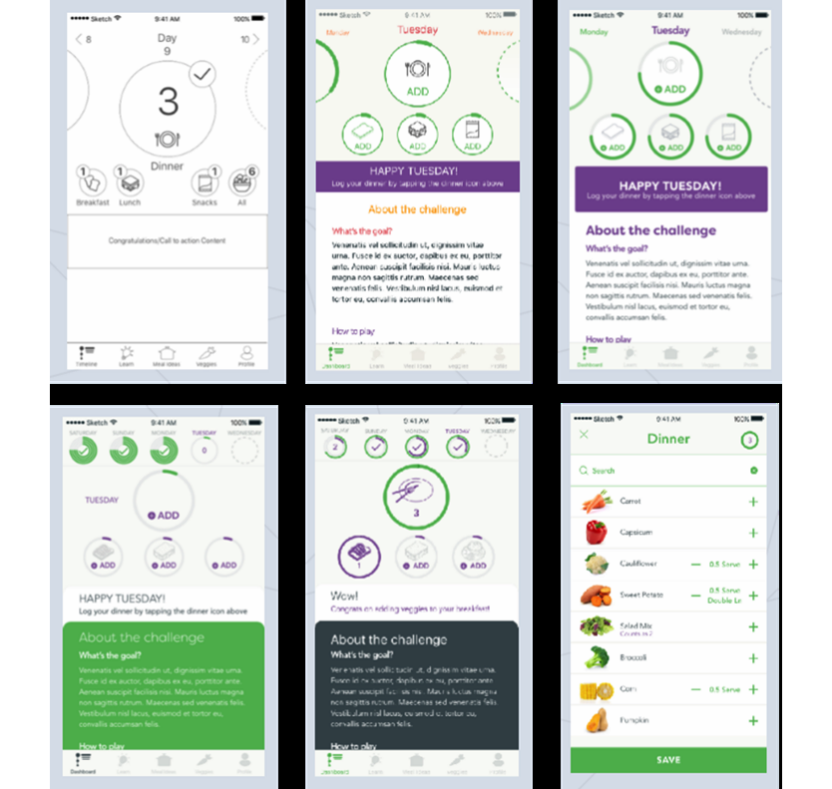
Early low resolution wireframes used in iterating the prototype for the vegetable tracker functionality of the app.

To empathize with users, the vegetable tracker feature needed to be quick and simple to use, highly applicable to a range of users by including a large variety of different vegetables and accommodating of users from across the health and motivation spectrums. Unlike traditional scientific methods, emphasis was therefore placed on simplicity and intuitiveness of this dietary intake recording tool. Its core functionality was to record variety of vegetables consumed (ie, the types) as well as servings (ie, the amount). A vegetable list of 125 vegetables was created, and a prototype vegetable tracker allowed users to scroll the vegetable list and click through to record the type and amount of vegetables consumed at each meal of the day. The prototype featured only the core component to ensure the app reached the target behavior as effectively as possible (Figure 2).

#### Phase 6: Gather User Feedback on the Prototype

The initial user testing provided feedback on the prototype and assessment of the functionality of navigating the vegetable list and logging vegetables consumed in meals. The key observations were that all participants were able to complete all tasks in minimal time and the flow of entering items was reasonably easily learnt. The meal icons for breakfast, lunch, dinner, and snacks appeared to be confusing, as users were not able to easily differentiate meals. Using circles for the meal icons also appeared to be confusing as users seemed to require more context in terms of what they were aiming for and how to fill them in. The recommendations for refinement from the development team included (1) further iteration on the design of meal icons, (2) creation of onboarding screens to help the user navigate, (3) more focus on the development of *progress indication*, and (4) to continue testing and iterating the dashboard design for further testing in the pilot trial. This feedback was included into the next iterations of the app.

#### Phase 7: Build Minimum Viable Product

The beta version of the app was developed with fully functional versions released and tested iteratively by the project team, with feedback provided to the project lead of the development team at regular intervals. The beta version of the app focused on the onboarding process, refining the vegetable tracker and user experience, developing a framework for providing feedback, and the visual branding.

**Figure 3:**
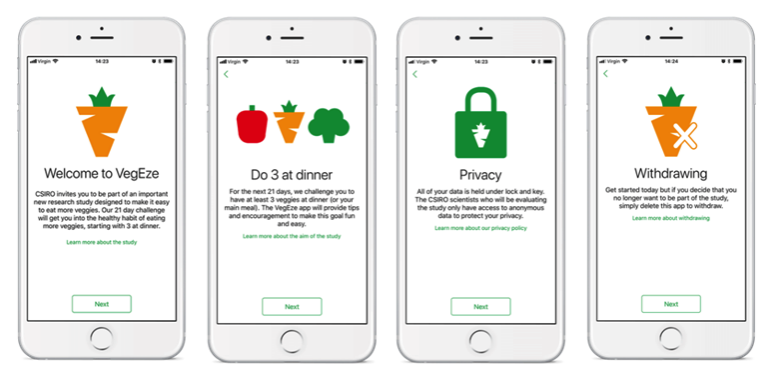
A sample of screenshots of the consent process which was embedded in the app using Apple ResearchKit software.

The onboarding process included ethics documentation and consent as well as the baseline evaluation survey. The consent process and evaluation surveys were embedded into the app using Apple ResearchKit software (Figure 3). The survey questions drew on previously validated questionnaires to assess amount and variety of vegetables (as the primary outcomes) [31] and psychological predictors of intake (as possible covariates of behavior change) such as attitudes, intentions, and nutrition-related self-efficacy [32-34]. The baseline evaluation survey was initially designed by researchers, and then refined for length and readability based on feedback from developers and the broader project team. Although it extends the registration process, inclusion of the survey is considered essential to allow for detailed evaluation of the app. Google Analytics was used to collect app usage data to allow us to understand the interactions between patterns of use and successful behavior change.

The user experience for the early versions of the app was centered on perceived ease of logging intake and usability of the vegetable list. In the prototype, the vegetable list was ordered by frequency of consumption per meal based on population intake data from the Australian National Nutrition Survey [35], and then over time ordered with user’s inputted data. Each vegetable item in the list had a name, image, and an information button (which allowed users to click through to details on standard serve size information). Serves could be added with a tap of the “ *+* ” and “ *−* ” icons for each vegetable in the list.

The framework for the frequency and content of feedback messages sent via push notifications was developed by the project team. A total of 3 types of notification messages would be sent including daily feedback for logging as well as content and recipe notifications which were sent on a random schedule 3 to 4 times per week. Daily feedback messages for logging intake and communicating progress were designed to be engaging and fun using friendly and positive language and emoji icons. Daily and weekly feedback and graphs on the variety and serves of vegetables logged were designed to be simple and informative and reinforce monitoring.

Finally, the creative team proposed various names and logos that were tested as part of the pilot study (phase 8). The name *VegEze* with the byline *Do 3 at dinner* received the most positive response from potential users and the project team agreed to use this branding for the app.

### Part 3: Assess Effectiveness and Usability

#### Phase 8: Pilot Potential Effectiveness and Usability

The majority of the participants who registered (492/553, 89%) reported they thought they would like to use the app at least once a day, and 87% felt they would use the app for 2 to 3 min per day or more. When asked how long they thought they could maintain the challenge of eating 3 different types of vegetables at dinner, 67% of users at baseline felt they could maintain it for 21 days or more.

#### App Usage

During the testing period (7 days), 265 (265/283, 94%) users who completed the baseline survey logged their vegetable intake at least once. Users averaged 5 sessions in the app during this period, with an average daily engagement time of 3 min 54 seconds. There were 1419 vegetable logs recorded, an average of 5.4 logs per user. The majority of these logs were for the evening meal (1042/1419, 73%).

#### Feedback Questionnaire

After downloading the app, at least 80% of users *agreed* or *strongly agreed* that the setup process of the app, including completing the baseline evaluation survey, was clear and relatively easy to complete. After using the app for a few days, 85% of users reported to like the tracking feature to record type of vegetables, and 79% liked the tracking feature to record serves consumed. Importantly, 75% of users felt the vegetable logging feature was easy to use, and 69% found it was useful:

A powerful motivator - more than I expected. I liked the quality of the images of vegetables and the search function.

I find the information about how much a serving size is for each vegetable is really useful. I would probably just assume I’m eating enough by having a few beans, pieces of carrot and broccoli florets for dinner - but know I know exactly how much I need to eat.

I like the scroll and search option to find veges and the incremental steps to measure vege intake. REALLY liked the option to click on the vege to show what a serving actually is AND the photo of the vege - great; ease of use, thumbs up.

Immediately following download, 75% of users (104/146) felt their overall experience of using the app was positive, and after a few days of using the app, 81% of users felt their experience was positive. Immediately following download, 86% of users reported they would use the app at least once a day, and this remained high at the end of the pilot trial (78% of users). Post download and post pilot, 80% and 87% of users, respectively felt they would use the app for 2 to 3 min. About one-third of users felt they would use the app for 1 to 3 months, and another third for more than 3 months. Finally, 63% of users who completed the post pilot survey (n=103) felt the app was easier or much easier than other health apps, and 69% indicated they would give the app 4 out of 5 stars

Enjoying using the app so far and it’s definitely challenged me to increase my veg intake at dinner.

Yes, I’m very impressed with how many veggies there are to choose from. Being a creature of habit it certainly opens your eyes to what you can eat.

#### Refinement Before Final Release

On the basis of the pilot evaluation, a few issues were identified by users in regard to the vegetable list and logging of vegetables that were addressed in the final sprint of the app. For example, the development team further refined the process for adding and deleting vegetables at different meal times to make it easier for users, the order of the vegetable list was revisited, and finally, users wanted more visual feedback of their total vegetable servings in addition to total types.

**Figure 4:**
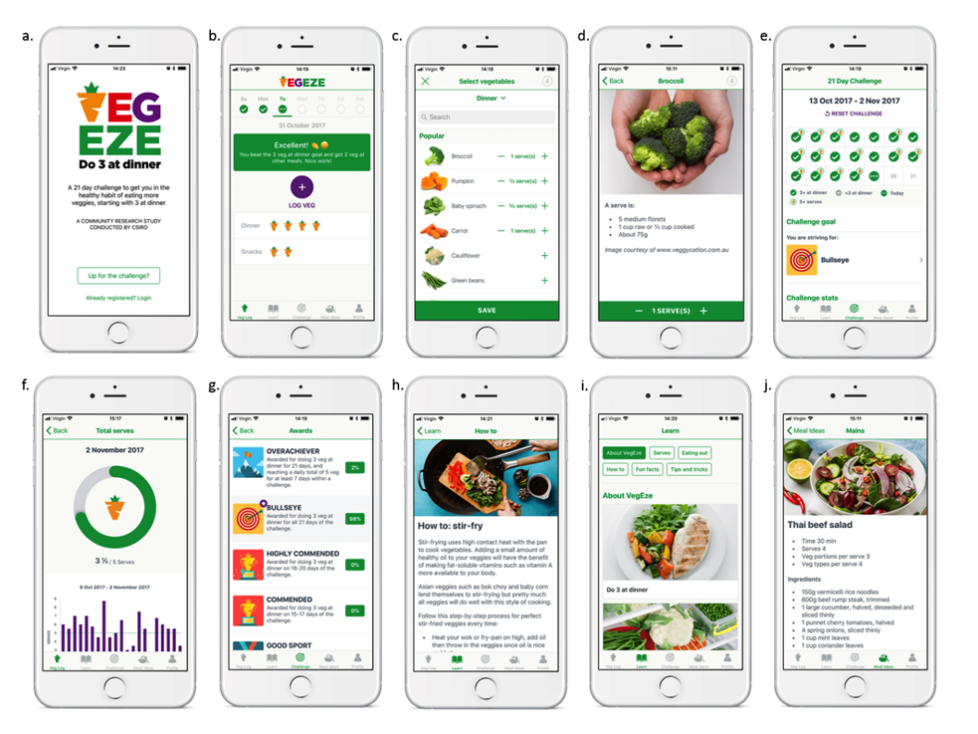
A sample of screenshots from the VegEze app as published in the App Store. (a) Log in and welcome screen; (b) Home screen displaying motivational feedback message, progress for current day for types of vegetables consumed, and vegetable log; (c) Vegetable list and logging functionality; (d) Example of a standard serve of vegetables; (e) Summary challenge screen and level achieved; (f) Feedback screen of serves of vegetables consumed for the day and across the challenge; (g) Leader board of achievements; (h) Example of “How to” content from the Learn section; (i) Learn screen; (j) Example of a recipe from the Meal ideas section.

The final sprint focused on the gamification elements of the app and the content, which was not part of the beta version. The consumer survey in phase 3 suggested that gamification features such as challenges and rewards were expected by 29% and 22% of respondents, respectively. Although these were not the most highly ranked features, gamification is increasingly popular within apps [26]. Gamification has the potential to facilitate behavior change by increasing motivation and making an everyday task more fun to achieve [25]. The release version of the app invites users to participate in a 21-day challenge to have 3 different types of vegetables at dinner (Figure 4). To encourage autonomy, challenges can be reset and restarted at any time by the user. In addition to the challenge, other gamification features include rewards and a form of leaderboard (Figure 4). A rewards-based scheme was built into the challenge whereby individuals achieved different levels depending on their intake. A leaderboard displays the different awards and the percentage of individuals using the app who are striving for each award—a deidentified form of social comparison. These gamification elements in the app were developed by the behavioral scientists and then further fleshed out by the project leaders to achieve features that were fun and motivating but also realistic within the project constraints.

The consumer survey described in phase 3 suggested that 82% of respondents expected recipe and meal ideas in the app as well as information on how to prepare vegetables (51% of respondents). The content framework divided articles into 3 categories: (1) evidence-based fun facts on vegetables, (2) recipe and meal ideas all containing 3 different vegetables, and (3) tips and tricks on how to prepare and include vegetables in meals (Figure 4). The recipe and meal ideas covered stir fries, mixed dishes, and main and side salads as well as meals with *hidden* vegetables given that the consumer survey suggested respondents were eating vegetables in a variety of ways. Moreover, 2 research dietitians worked together to develop 74 short articles and 57 recipes and meal ideas for inclusion in the app.

Two-way user feedback was central to the app (Figure 4). The home screen became the place where users can review their progress of the day with carrot icons representing the number of types consumed in each meal for the current day. With a bar slider at the top of the screen, users can review previous days at a glance with a *tick* indicating that the goal was met (Figure 4) or across the challenge period in a calendar format (Figure 4). With a swipe from the home screen, users can also review their progress toward reaching the recommended number of serves per day as well as review the past progress on reaching this target with a simple visual bar chart (Figure 4).

As described in phase 2, the app drew on theory-based BCTs. The final release version of the app was independently coded by 2 trained research assistants, who identified 16 BCTs used within the app, most of which were from the goals and planning subsections of the BCT taxonomy (Table 4).

**Table 4:** The VegEze app was coded for the inclusion of 16 behavior change techniques (BCTs) from the BCT taxonomy.

BCT category		BCT description	Example of intervention component in the app
**Goals and planning**			
	1.1 Goal setting (behavior)	Set or agree a goal defined in terms of the behavior to be achieved	Sets a goal to eat 3 types of vegetables at dinner each day for 21 days
	1.4 Action planning	Prompt detailed planning of performance of the behavior	Encourages planning and preparation to eat a variety of vegetables at dinner
	1.5 Review behavior goals	Review behavior goals in light of achievement	Daily feedback messages and visual displays provided on types of vegetables consumed
	1.6 Discrepancy between current behavior and goal	Draw attention to discrepancies between current behavior and the goal	Home screen and daily feedback messages point out differences between the number of types of vegetables consumed and target; and feedback screen provides feedback about consumption of serves of vegetables relative to Dietary Guidelines
	1.7 Review outcome goals	Review the outcome goal in light of achievement	At the end of the challenge, feedback on achievement is provided and a new goal can be set
**Feedback and monitoring**			
	2.2 Feedback on behavior	Monitor and provide informative or evaluative feedback on behavior	Daily feedback messages and visual displays provided on types of vegetables consumed
	2.3 Self-monitoring of behavior	Establish a method for the person to monitor and record their behavior	Vegetable log asks participants to record consumption daily
**Natural consequences**			
	5.1 Information about health consequences	Provide information about health consequences of behavior	Learn section provides information on the health benefits of consuming vegetables
	5.3 Information about social and environmental consequences	Provide information about social and environmental consequences of behavior	Learn section provides information about the benefits to their family and environmental benefits to consume and not waste vegetables
	5.6 Information about emotional consequences	Provide information about emotional consequences of behavior	Learn section provides information that eating vegetables increases energy and sense of well-being
**Comparison of behavior**			
	6.2 Social comparison	Draw attention to other people’s performance to allow comparison with own performance	A leaderboard displays the different awards and the percentage of individuals using the app who are striving for each award
**Repetition and substitution**			
	8.1 Behavioral practice or rehearsal	Prompt practice of the behavior to increase habit	Daily push notifications to prompt behavior
	8.3 Habit formation	Prompt rehearsal and repetition of the behavior in the same context repeatedly	Push notification to prompt consumption of 3 types at dinner each day
**Reward and threat**			
	10.3 Nonspecified reward	Arrange delivery of a reward if there have been effort and progress in behavior	Virtual rewards received for logging and achieving progress toward 3 vegetables at dinner
	10.6 Nonspecific incentive	Inform that a reward will be delivered if effort and progress in behavior are made	Information section informs of virtual rewards for logging and achieving progress toward 3 vegetables at dinner
**Scheduled consequences**			
	14.5 Rewarding completion	Build up behavior by arranging reward following final component of the behavior	Participants receive virtual rewards for eating 3 vegetables, which is contingent on them buying, cooking, and eating vegetables

#### Phase 9: Evaluate With a Trial of the Product

It is planned that the overall impact of the VegEze app will be evaluated using the Reach, Effectiveness, Adoption, Implementation, and Maintenance (RE-AIM) framework [36,37] in an uncontrolled, quantitative study designed to measure its effectiveness in increasing daily vegetable consumption after 21 and 90 days as well as to determine the associations between user characteristics and outcome measures. The RE-AIM framework focuses the evaluation on 5 program components that are considered together to determine the public health impact of the initiative. These components include reach, effectiveness, adoption, implementation, and maintenance. The RE-AIM evaluation framework is commonly used to translate research and understand impact within *real-world* settings [36,37]. Changes in vegetable intake will be measured via self-report in the surveys embedded in the app and will be administered at baseline, day 21, and day 90. User characteristics, which will be explored in the evaluation trial for their association with app usage patterns or dietary intake, include, for example, demographic characteristics such as age and gender, health indicators such as weight status, and baseline vegetable intake.

VegEze is currently available in the App store as a standalone research app, and participants have been recruited to take part in the evaluation study through television, radio, and social media. The results of this evaluation will be published and the execution of a communication plan will ensure the results are shared widely at the completion of the trial (phase 10 of the IDEAS framework but not discussed here).

## Discussion

### Overview

This paper has described the theoretically and commercially derived development of the VegEze app and its features and functionality. Combining a theoretical framework with an industry perspective and input has resulted in an app that was developed in a timely manner while retaining its evidence-base. It has been suggested that the pace of the development, evaluation, and dissemination cycle is too structured and slow in academia, inhibiting the progress in meaningful and engaging smartphone interventions [12,17]. However, few have worked closely with industry partners to remedy this. The quick build phase of VegEze is evidence that academic-industry partnerships can work efficiently and effectively to develop products that target important population health issues.

There are advantages and disadvantages of a rapid build phase that should be acknowledged. The sequential sprints and the staged release of new app features at the completion of each sprint allowed the project team to provide timely feedback to the development team in a highly iterative and continuous refinement process. The rapid development phase also ensured the project team focused on the essential functionality and core features expected by the target users and spent relatively less time on the *nice to have* features that were of limited appeal and utility to our audience. However, with more time we may have been able to benchmark normal usage and develop strategies to maximize user engagement for all features of the app. Regardless, this project demonstrates that a high-quality app that meets users’ expectations can be built in a short time frame, with industry support, a commercial development partner, and a motivated multidisciplinary project team.

Internet and mobile phone technology are commonly used as a delivery medium to promote health behavior, both in research and within the health and wellness industry. It has been estimated that half of all mobile phone users have downloaded a health-related app, with fitness and nutrition apps being most commonly downloaded and used at least once daily [38]. However, the effectiveness of these products is not always evaluated, and more specifically, the features of digital products that are associated with effective behavior change is not well understood [14,15]. Only a small proportion of commercially available apps have a theoretical grounding [15], yet reviews of digital interventions published in the scientific literature support the use of theoretical frameworks and theoretically based BCTs [14,15]. A recent review suggested interventions that used theory more extensively and included a greater number of BCTs were more effective than those without a strong theoretical foundation or which used fewer techniques [14]. VegEze is available in the App store; however, it differs from other commercially available apps—its development was strongly guided by an evidence-based framework, behavioral theory, and drew on 16 BCTs. The planned trial will evaluate the impact of VegEze and explore the use of app features and their association with increased vegetable intake.

Review of literature suggest interventions in the general adult population can increase consumption of fruits and vegetables by 0.2 to 0.6 serves per day or up to 1.4 in more controlled environments [39], but this increase is likely because of changes in fruit intake and to a lesser extent, vegetable intake [40]. A more recent review of electronic and mobile health interventions for young adults found from the studies that reported vegetable intake independent of fruit, 4 out of 5 studies increased intake, with reported increases between 0.1 and 0.4 serves per day [41]. Through media coverage and the use of social media, we are expecting that the VegEze app will reach over 5000 people and achieve an average increase of between a quarter to half a serve, and possibly higher in those who have low vegetable intakes.

### Strengths

Self-monitoring is one of the most commonly used BCT within smartphone interventions [15]. The VegEze app has at its core the fundamental behavioral techniques of self-monitoring and goal setting. These techniques have been applied in interventions targeting a multitude of behaviors and are consistently identified in various theories as fundamental for the process of behavior change [42]. However, how these are operationalized is critical to the target behavior. We have been careful in how these techniques have been applied in a way tailored specifically to the behavior of interest. We have also been careful in balancing the scientific evidence-base, with the fun, engaging, and usability elements expected from commercial grade apps—largely the result of an ongoing exchange of ideas between the members of the multidisciplinary project team. Finally, the IDEAS framework helped to focus the priorities of the app development and retain scientific method. The use of Apple ResearchKit software means that evaluation surveys are embedded into the app and will allow for a robust evaluation phase. Using the ResearchKit software also saved on development time; however, restricting the initial build to Apple products could also be seen as a limitation. The results and feedback from the planned evaluation study will inform further development of the iOS app as well as expansion to Android products.

### Limitations

Although the development of the app was informed by large groups of potential users, they were individuals who had previously registered or participated in health-related initiatives and are likely to have a higher vegetable intake overall than the general population. The barriers to consumption and strategies to increase intake may differ in those who are already consuming vegetables compared with those with low consumption or compared with those who intend to increase consumption. The appeal and impact of the app to a broader range of users will be part of the evaluation. In addition, the majority of the consumer sample were women, aged in their late 40s or early 50s, health motivated, and all volunteered to help in the development of an app targeting vegetable consumption. It is possible that the particular nature of this sample may have informed the development of app features that do not appeal as much to other groups of the population. We expect using mass and social media to recruit for the evaluation trial will result in a more diverse group of users so that as part of the evaluation we can better understand the variation in uptake and usage of the app by different user characteristics. Participants of the pilot trial used the app for 5 out of a possible 7 days, with an average daily engagement time of almost 4 min. Another literature suggests that, among mobile phone users in the United States, 65% opened their health apps once per day, and 44% used their app for between 1 and 10 min [38]. Achieving sufficient engagement and maintaining user retention rates is difficult, and although the usage of participants in the pilot trial was similar to other research, we will explore usage patterns in greater detail as part of the planned evaluation trial.

### Conclusions

The development of VegEze was the result of a research-industry partnership that brought together scientific evidence and commercial *know how* to develop an app targeting inadequate vegetable consumption among Australian adults. The IDEAS framework involved a number of iterative steps and helped to retain a theoretical foundation without compromising the pace of the pathway to market. The effectiveness of the app is currently being evaluated in a large-scale, *real-world* trial, and the results will be reported using the RE-AIM framework.

## Abbreviations

BCT: behavior change techniques
BCW: behavior change wheel
COM-B: capability, opportunity, and motivation to engage in the behavior
CSIRO: Commonwealth Scientific and Industrial Research Organisation
IDEAS: integrate, design, assess, and share
RE-AIM: reach, effectiveness, adoption, implementation, and maintenance
